# Let-7c-3p Regulates Autophagy under Oxidative Stress by Targeting ATG3 in Lens Epithelial Cells

**DOI:** 10.1155/2020/6069390

**Published:** 2020-03-16

**Authors:** Ting Li, Yanhong Huang, Wenkai Zhou, Qichang Yan

**Affiliations:** ^1^Department of Ophthalmology, The Fourth Affiliated Hospital of China Medical University, Shenyang, China; ^2^Department of Ophthalmology, Shenyang Women's and Children's Hospital, Shenyang, China

## Abstract

**Background:**

Oxidative stress is an important factor during age-related cataract formation. Apoptosis and autophagy induced by oxidative stress have been reported as key factors in age-related cataract. In our research, we investigated the role of let-7c-3p in the regulation of autophagy and apoptosis during the formation of age-related cataract. *Material and Methods*. Real-time PCR and western blot were employed to detect the expression of let-7c-3p in the tissues of age-related cataract. Human lens epithelial cells (LECs) were treated with H_2_O_2_ as an age-related cataract model. The extent of apoptosis was measured by flow cytometry and western blot. To detect autophagy, immunofluorescence was used to analyze the spot number of LC3, and western blot was used to detect the expression of LC3-II/I and ATG3. The molecular mechanisms of let-7c-3p regulating autophagy via ATG3 under oxidative stress were performed by a luciferase report gene assay and rescue experiment.

**Results:**

Downregulation of let-7c-3p was found in the age-related cataract group aged >65 years relative to the age-related cataract group aged ≤65 years. Consistently, the expression of let-7c-3p was also lower under oxidative stress. The activities of LEC apoptosis and autophagy induced by oxidative stress were inhibited by let-7c-3p. By the bioinformatics database and the luciferase reporter assay, ATG3 was found to be a direct target of let-7c-3p. Let-7c-3p reduced the ATG3-mediated autophagy level, which was induced by oxidative stress in LECs.

**Conclusion:**

Let-7c-3p inhibits autophagy by targeting ATG3 in LECs in age-related cataract.

## 1. Introduction

Globally, cataract is a prior cause of blindness and aging is the leading contributor of the cataracts [1]. Ultraviolet [2] and oxidative stress [3] are seen as key factors in cataract formation. A meaningful relationship [4] has been revealed between age-related cataract and oxidative stress. Oxidative stress refers to the damage of exogenous or endogenous ROS beyond the antioxidant capacity of cells, which affects the signal transduction system of cells or subsequently damages macromolecules such as nucleic acids, proteins, and lipids [5]. Oxidative stress plays a critical role in regulating normal physiological functions associated with cell cycle [6], migration [7], and cell death [8].

In previous study, oxidative stress has also been declared to induce apoptosis [9] and autophagy [10] in lens epithelial cells (LECs). There are many studies on the potential mechanism of new targets in the treatment of age-related cataract based on autophagy and apoptosis [11]. Some antioxidative agents can decrease H_2_O_2_-treated human lens epithelial cell damage; Ma et al. show that HO-1 can protect human lens epithelial cells from H_2_O_2_-induced oxidant stress by upregulating antioxidant enzyme activity, reducing ROS generation, and thus inhibiting caspase family-dependent apoptosis. And Morishita et al. found that deletion of autophagy-related 5 (Atg5) and Pik3c3 genes in the lens causes cataract independent of programmed organelle degradation [12]. However, the molecular mechanisms during the process of age-related cataract formation remain unclear.

Overaccumulation of ROS destroys the homeostasis of cells, while autophagy reduces oxidative damage by phagocytosis and degradation of irreversible oxides. Autophagy is a highly conserved process involving protein, lipid, and organelle degradation, which results in influencing nutrition recycle [13]. When LC3-II is measured as an autophagy-related marker, the increase of the LC3-II/LC3-I ratio represents the active autophagy activity [14]. Almost all cells experience basal autophagy to maintain cellular homeostasis. But when under stress conditions, such as hypoxia and starvation, autophagy will be switched on to protect cells from these stress conditions [15]. Dysregulation of autophagy has been reported to be related to various diseases [16]. In addition, sometimes, the role of autophagy in disease progression is dual. A recent study has found that autophagy can act a very important role in keeping the transparency of lenses [17]. Moreover, autophagy can also participate in regulating pathophysiologic process in hereditary cataract [18] and age-related cataract [19]. It has been found that autophagy is scheduled to be constitutively activated in lens epithelial cells during the period of fiber cell differentiation [20]. It is very likely that autophagy is important for protecting lens cells from oxidative stress, which is one of the main causes of age-related cataract [21].

A growing number of studies have indicated that autophagy could be controlled by microRNAs (miRNAs) [22, 23]. miRNAs are a number of small noncoding RNAs with 20~24 nucleotides that inhibit gene expression, such as mRNA degradation and translational inhibition, by binding the 3′UTR region of mRNA [24]. miRNAs have been recorded to regulate cell proliferation, metastasis [25], apoptosis [22, 26], and autophagy [23]. Recently, the role of miRNAs in age-related cataract has been noted. And miR-326 inhibitor increased *β*B2 expression via upregulated FGF1, which may influence the progression of age-related cataract [22]. The bcl-2 protein is located in the endoplasm momentum and mitochondrial membranes, and it can inhibit the release of apoptosis-inducing factors to prevent cell apoptosis. In apoptosis, Bax protein activates the cascade of reactions by releasing cytochrome c from the mitochondria that helps in successive activation of caspases and ultimately leads to cell death [27]. Let-7b was noted to induce apoptosis of LECs through targeting leucine-rich repeat containing G protein-coupled receptor 4 (Lgr4) [26]. The miRNA let-7c has been tried and found downregulated in cataract [28]. In mammals, let-7 is known as the keeper of differentiation, and its abnormal regulation and expression have been associated with disease progression [29]. The let-7 miRNA family was proven to inhibit the cellular reprogramming process. Reprogramming well-differentiated cells into induced persistent stem cells had a great significance on tissue repair and tumor occurrence [30]. Recently, some findings suggest that microRNAs have a role in age-related cataracts. A local let-7 microRNA increase may represent a risk factor in the formation of age-related cataracts [31]. The aim of our study is to research the differentially expressed let-7c in age-related cataract whether it can influence autophagy, a fundamental degradation process in LECs, which can improve our understanding of cataract.

In the current study, we explored the function of let-7c-3p in the regulation of autophagy in LECs during age-related cataract formation. We detected the expression of let-7c-3p with age-related cataract tissues and LEC cells treated with hydrogen peroxide. The effect of let-7c-3p on autophagy and apoptosis was evaluated under oxidative stress. Moreover, we investigated molecular mechanisms of let-7c-3p regulating autophagy in LECs. We confirmed that let-7c-3p regulates autophagy by targeting ATG3 under oxidative stress in lens epithelial cells.

## 2. Material and Methods

### 2.1. Clinical Specimens

A total of 40 anterior lens capsules were collected at the Fourth Affiliated Hospital of China Medical University from age-related cataract patients undergoing phacoemulsification surgery (patients were excluded if they were affected by other eye diseases). In total, 20 of the samples were collected from males and 20 from females. All samples were divided into two groups, aged 66-78 (70.23 ± 4.41) and 53-65 (58.23 ± 3.03). The study was approved by the hospital ethics committee. All patients provided written informed consent to use the research of tissue samples.

### 2.2. Cell Lines and Treatment

Human lens epithelial cells (SRA01/04 lines) (ATCC, USA) were cultured in Dulbecco's modified Eagle's medium (DMEM) supplemented with 10% fetal bovine serum and 100 U/mL penicillin-streptomycin solution in a humidified 5% CO_2_ incubator at 37°C. Let-7c-3p mimics, mimic control, let-7c-3p inhibitor, inhibitor control (RiboBio Co. Ltd, Guangzhou, China), ATG3 plasmid, and pcDNA3.1 plasmid (Invitrogen, USA) were transfected into SRA01/04 cell using Lipofectamine 3000 (Invitrogen, USA) as described in the manufacturer's instructions.

### 2.3. Cell Viability

SRA01/04 cells were cultured in 96-well microplates with a density of 1 × 10^4^ cells/well and incubated with multiple concentrations (0–150 *μ*M) of H_2_O_2_ (6 h). After 24 h incubation, the cells were obtained, and cell viability was then measured by a CCK8 assay.

### 2.4. Real-Time PCR and RNA Interference

Total RNA in anterior lens capsules and cells was extracted with TRIzol reagent. A TaqMan™ microRNA kit was employed to obtain miRNA cDNAs. The expression of let-7c-3p was analyzed by a TaqMan microRNA kit. RNA reverse transcription was performed with a retrovirus PrimeScript™ kit. TaqMan Universal Master Mix II kits were used to detect the ATG3 mRNA expression level and *β*-actin as inner reference. All primers were as follows: let-7c-3p forward 5′-CTGATTTGGACAAGCAGCAA-3′ and reverse 5′-CTGGACAAACCTCAGCCCTA-3′, ATG3 forward 5′-GACCCCGGTCCTCAAGGAA-3′ and reverse 5′-TGTAGCCCATTGCCATGTTGG-3′, let-7c-3p forward 5′-GCGCGTGAGGTAGTAGGTT-3′ and reverse 5′-GTGCAGGGTCCGAGGT-3′, and *β*-actin forward 5′-AAAGATGTGCTTCGAGATGTGT-3′ and reverse 5′-CACTTTGTCAGTTACCAACGTCA-3′. The primer of let-7c-3p was designed by Takara (Dalian, China). The sense sequences of the ATG3 small interfering RNA were synthesized by GeneChem corporation (Shanghai, China) as follows: siRNA 5′-CCCAGAAGAGUUUGUGGCAGCUGGA-3′.

### 2.5. Western Blot

The samples were homogenized in buffer containing RIPA and phenylmethylsulfonyl fluoride (Roche, Nutley, NJ, USA). Protein expression in HCC cell lines was detected using immunoblotting. The polyvinylidene difluoride membrane was used to transfer the protein after protein was loaded onto a sodium dodecyl sulfate polyacrylamide gel electrophoresis mini gel. Rabbit polyclonal primary antibody (Abcam, Cambridge, MA) was used to incubate the membranes. And then, horseradish peroxidase-conjugated secondary antibody was employed. Subsequently, enhanced chemiluminescence substrates (Millipore, Billerica, MA) were obtained to visualize the signals. Glyceraldehyde 3-phosphate dehydrogenase was utilized as an endogenous protein for normalization. The homogenates were tested on polyacrylamide gels, moved onto PVDF membranes (Thermo Fisher Scientific, Billerica, USA), and then probed with specific primary antibodies. Western blot was used to detect the expression of LC3-II/I, ATG3, and apoptosis-related protein.

### 2.6. Assessment of Apoptotic Cells

The extent of apoptotic cells was analyzed by an Annexin V-FITC apoptosis detection kit (Beyotime Institute of Biotechnology) following the manufacturer's instruction. Briefly, the cells were harvested, washed with phosphate-buffered saline, and resuspended using 500 *μ*L binding buffer. Next, 5 *μ*L Annexin V-FITC and 5 *μ*L propidium iodide (PI) were supplemented to the buffer. Cell apoptosis was analyzed by FACSCalibur flow cytometry (BD Bioscience). The fraction of the cell amount was measured by quadrant statistics.

### 2.7. Immunofluorescence Microscopy

SRA01/04 cells were seeded on coverslips and fixed with 4% paraformaldehyde for 25 minutes at room temperature after various treatments. The primary antibody (1 : 100; LC3#PM036) was added and incubated at 4°C overnight. After washing for three times (5 mins/time), the fluorescent secondary antibody (1 : 100; FITC#A22120) was added and incubated for 45 minutes at 37°C. Finally, the cells were stained with diaminophenylindole (Beyotime; #C1002) and visualized by a confocal microscope (Nikon A1r). Immunofluorescence was used to analyze the spot number of LC3.

### 2.8. Luciferase Reporter Assays

The 3′UTR of ATG3 containing the putative target site for let-7c-3p and the mutant sequences were amplified by PCR and inserted into the pmiR-RB-REPORT (RiboBio). SRA01/04 cells were transfected with the wt ATG3-3′UTR or mut ATG3-3′UTR, let-7c-3p mimics, mimic control, let-7c-3p inhibitor, and inhibitor control by Lipofectamine 3000 (Invitrogen). After transfection for 48 h, luciferase activity was analyzed by the dual-luciferase assay system (Promega).

### 2.9. Statistical Analysis

All experiments were performed three times. All results are expressed as means ± SD unless indicated otherwise. Statistical analysis was carried out using the Student *t*-test or one-way analysis of variance. Differential expression was analyzed by SPSS software (Version 17.0, Chicago, IL, USA). *P* value < 0.05 was considered statistically significant.

## 3. Results

### 3.1. The Expressions of Let-7c-3p Were Downregulated in Cataract Tissues and LECs

**Figure 1:**
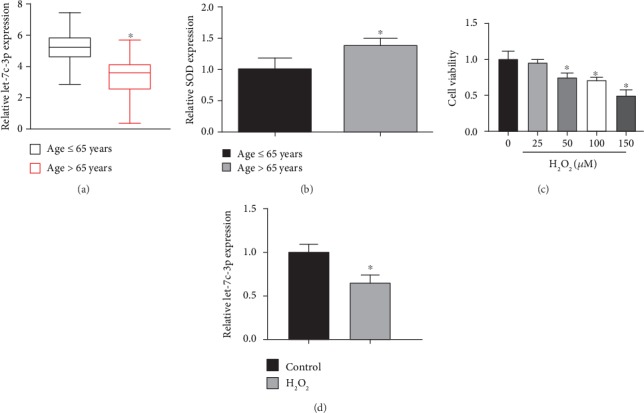
The expressions of let-7c-3p were downregulated in the anterior lens capsules and in SRA01/04 cells under oxidative stress. (a) Let-7c-3p expression levels in samples with age > 65 years relative to samples with age ≤ 65 years were detected by real-time PCR. (b) Let-7c-3p expression levels in samples with age > 65 years relative to samples with age ≤ 65 years were detected by real-time PCR. (c) The cell viability in SRA01/04 cell treated with a series of H_2_O_2_ concentrations (0-150 *μ*M). CCK8 assay was used to detect the levels of cell viability. (d) Let-7c-3p expression level in SRA01/04 cells under oxidative stress was detected by real-time PCR (^∗^*P* < 0.05).

As shown in the previous research [31], age is significantly correlated with the severity of cataract. Therefore, we divided the cataract patients into two groups according to age. Real-time PCR analysis of let-7c-3p expression showed that the level of let-7c-3p in anterior capsules of age-related cataract patients (age > 65 years) was significantly lower than that in anterior capsules of age-related cataract patients (age ≤ 65 years) (Figure 1(a)). And we found that the SOD expression level was higher in the age > 65 years group than the age ≤ 65 years group. Oxidative stress is one of the crucial factors of age-related cataract [4]. Therefore, we examined the cell viability in SRA01/04 cells treated by various concentrations of H_2_O_2_. The results showed that 50 *μ*M H_2_O_2_ condition was set as work concentration (Figure 1(c)). SRA01/04 cells were treated with 50 *μ*M H_2_O_2_ for 24 h. Real-time PCR was then used to detect let-7c-3p expression. We demonstrated that the level of let-7c-3p expression in lens epithelial cells under oxidative stress was significantly lower than that of the normal group (Figure 1(d)). This indicated that let-7c-3p was downregulated in SRA01/04 cells under oxidative stress.

### 3.2. Let-7c-3p Attenuated the Apoptosis in SRA01/04 Cells under Oxidative Stress

**Figure 2:**
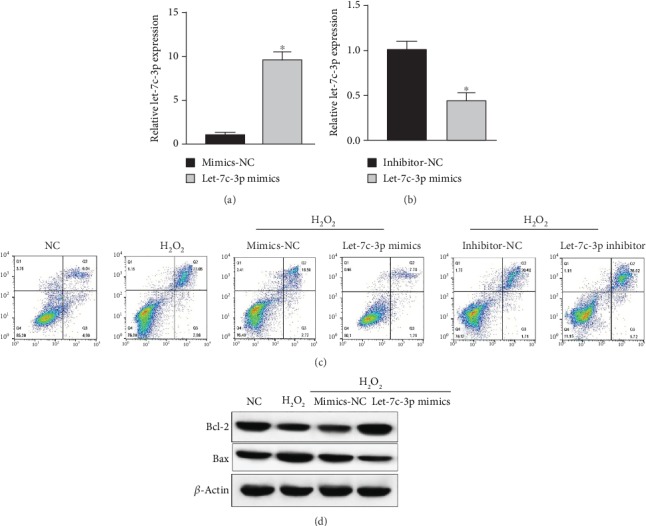
Let-7c-3p attenuated the apoptosis in SRA01/04 cells under oxidative stress. (a, b) The mRNA expression levels of let-7c-3p in SRA01/04 cells infected by let-7c-3p mimics, mimic controls, let-7c-3p inhibitors, or inhibitor controls were detected by real-time PCR. (c) Forty-eight hours after infection, downregulated let-7c-3p and control groups were treated with 50 *μ*M H_2_O_2_ for 24 h. Flow cytometry was used to analyze apoptosis. (d) Western blot was used to analyze the expression level of Bcl-2 and Bax. The SRA01/04 cells were treated with 50 *μ*M H_2_O_2_ for 24 h (^∗^*P* < 0.05).

To explore the effect of let-7c-3p on apoptosis under oxidative stress, LECs were infected with let-7c-3p mimics and let-7c-3p inhibitors, respectively. The transfection efficiency was analyzed by real-time PCR (Figures 2(a) and 2(b)). We observed that the apoptosis rate of SRA01/04 cells was induced by oxidative stress (Figure 2(c)). The rate of apoptosis in SRA01/04 cells increased from 6.04% to 22.50%. Meanwhile, the rate of LEC apoptosis decreased from 18.50% to 7.70% when SRA01/04 cells were infected by let-7c-3p mimics compared to the negative control. And the rate of LEC apoptosis decreased from 20.40% to 26.02% when SRA01/04 cells were infected by let-7c-3p inhibitor compared to the negative control. To further confirm this result, we analyzed Bcl-2 and Bax protein expression and found that the results were consistent with flow cytometry (Figure 2(d)). These results implied that let-7c-3p attenuated apoptosis under oxidative stress.

### 3.3. Let-7c-3p Attenuated the Autophagy in SRA01/04 Cells under Oxidative Stress

**Figure 3:**
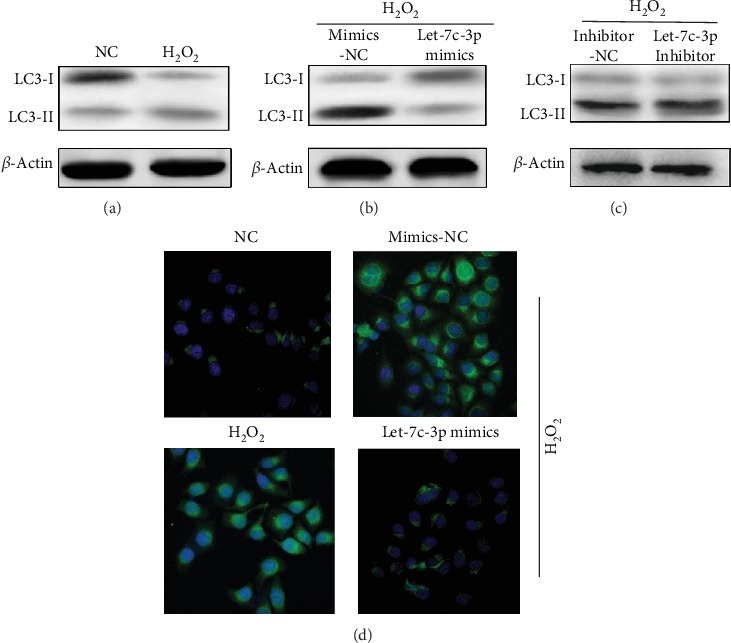
Let-7c-3p attenuated the autophagy in SRA01/04 cells under oxidative stress. (a) Western blot was used to analyze the expression level of LC3B II and LC3B I proteins in SRA01/04 cells infected by let-7c-3p mimics, let-7c-3p inhibitor, and mimic controls under oxidative stress. (b) The effect of enhanced let-7c-3p on LC3 puncta in SRA01/04 was explored by immunofluorescence. The SRA01/04 cells were treated with 50 *μ*M H_2_O_2_ for 24 h (^∗^*P* < 0.05).

As autophagy and apoptosis both participate in formation of cataract, we tried to investigate whether let-7c-3p could modulate autophagy. SRA01/04 cells were exposed to oxidative stress as an experiment group for 24 h and then treated with let-7c-3p mimics and let-7c-3p inhibitor. Under oxidative stress, we observed that the ratio of LC3B II and LC3B I proteins increased significantly in SRA01/04 cells, while the ratio decreased when LECs were transfected by let-7c-3p mimics compared with the control group. However, the let-7c-3p inhibitor could increase the ratio of LC3B II and LC3B I (Figure 3(a)). To further investigate the effect of let-7c-3p on autophagy, an immunofluorescence assay was conducted. The result showed that let-7c-3p could suppress autophagy induced by H_2_O_2_ (Figure 3(b)). Thus, the findings suggested that let-7c-3p attenuated the level of autophagy in SRA01/04 cells under oxidative stress.

### 3.4. ATG3 Facilitated Autophagy in SRA01/04 Cells under Oxidative Stress

**Figure 4:**
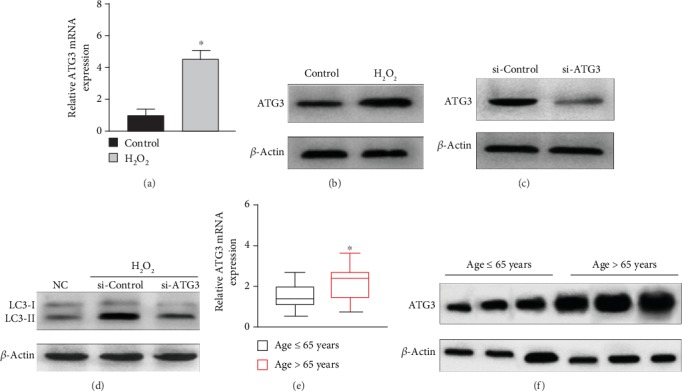
ATG3 attenuated the autophagy in SRA01/04 cells under oxidative stress. (a, b) Western blot and real-time PCR showed the mRNA and protein expression level of ATG3 in SRA01/04 cells treated with 50 *μ*M H_2_O_2_ for 0 h and 24 h. (c) Real-time PCR showed the transfection efficiency of ATG3. (d) Western blot was used to analyze the ratio of LC3B II and LC3B I proteins in SRA01/04 cells treated by si-ATG3 and NC group under oxidative stress. (e) The expression of ATG3 mRNA in cataract tissues. (f) The expression of ATG3 protein in cataract tissues was analyzed by western blot (^∗^*P* < 0.05).

We conducted real-time PCR and western blot assays to detect the expression of ATG3 in SRA01/04 cells under oxidative stress. We found that ATG3 was upregulated in SRA01/04 cells under oxidative stress (Figures 4(a) and 4(b)). ATG3 has been reported as a vital modulator of autophagy in mediating mitochondrial homeostasis [32]. To confirm the effect of ATG3 in LECs, we performed the loss-of-function study. After transfection for 24 h, the level of ATG3 was downregulated by si-ATG3 (Figure 4(c)). We found that the ratio of LC3B II and LC3B I proteins in the si-ATG3 group was lower than that in the negative control (Figure 4(d)). These findings revealed that ATG3 facilitated the autophagy in SRA01/04 cells under oxidative stress. In addition, ATG3, an E2-like enzyme, is essential for vesicle elongation formation and plays a significant role in autophagy regulation. Then, we detected the expression level of ATG3 in cataract tissues. The results showed that ATG3 in patients aged >65 years was higher than that in the patients aged ≤65 years (Figures 4(e) and 4(f)).

### 3.5. Let-7c-3p Regulates Autophagy by Targeting ATG3 in SRA01/04 Cells under Oxidative Stress

**Figure 5:**
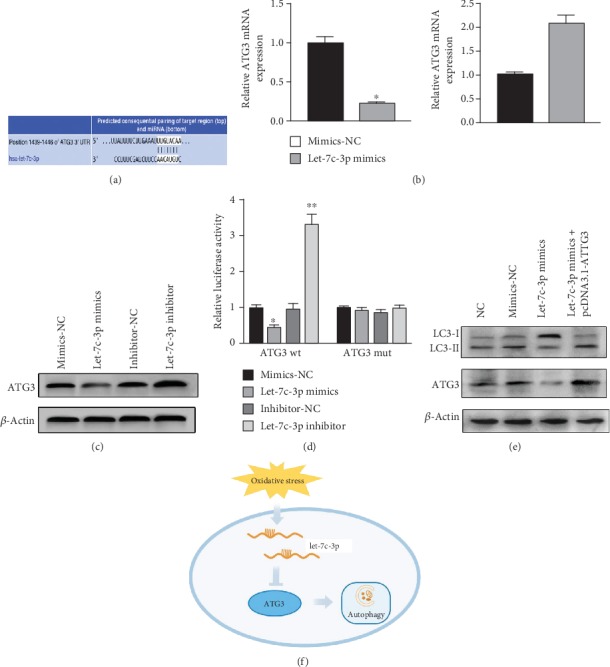
Let-7c-3p regulates autophagy by targeting ATG3 in SRA01/04 cells under oxidative stress. (a) Putative let-7c-3p binding site in the 3′UTR region of ATG3. (b, c) The expression levels of ATG3 mRNA and protein regulated by upregulated or downregulated let-7c-3p. Real-time PCR and western blot were used to detect the level of ATG3 mRNA and protein expression. ATG3 expression was downregulated when SRA01/04 cells were transfected with let-7c-3p mimics and upregulated with let-7c-3p inhibitors. (d) Relative luciferase level among wild-type ATG3-3′UTR, mutated ATG3-3′UTR, mimics-NC, let-7c-3p mimics, inhibitor-NC, and let-7c-3p inhibitor. LECs were transfected with let-7c-3p mimics, mimics-NC, inhibitor-NC, let-7c-3p inhibitor, wild-type 3′UTR, and mutated 3′UTR of ATG3. Luciferase activity was analyzed after 48 h. (e) Western blot was used to analyze the expression level of LC3B II and LC3 I proteins in SRA01/04 cells infected by mimic controls, let-7c-3p mimics, and let-7c-3p mimics with pcDNA3.1-ATG3 under oxidative stress. (f) The schematic of let-7c-3p-regulating autophagy by targeting ATG3 in SRA01/04 cells under oxidative stress (^∗^*P* < 0.05).

The prediction by the TargetScan database (http://www.targetscan.org/vert_72/) indicated that ATG3 might be the target gene of let-7c-3p (Figure 5(a)). To indicate the prediction result, we use real-time PCR and western blot to measure ATG3 expression levels after transfecting with let-7c-3p mimics and let-7c-3p inhibitors in SRA01/04 cells. The results showed that ATG3 mRNA and protein expression increased when let-7c-3p was downregulated, while the levels of ATG3 mRNA and protein expression decreased when let-7c-3p was upregulated (Figures 5(b) and 5(c)). Consistently, the luciferase reporter assay was performed, showing that let-7c-3p could bind with ATG3 mRNA directly (Figure 5(d)). To examine whether let-7c-3p could regulate autophagy via ATG3, we performed a rescue experiment. The results showed that ATG3 reversed the effect of let-7c-3p on attenuating the autophagy level (Figure 5(e)). In conclusion, let-7c-3p could regulate autophagy by targeting ATG3 in SRA01/04 cells under oxidative stress (Figure 5(f)).

## 4. Discussion

Since aging predominates in the formation of cataracts [1], we performed real-time PCR and western blot in various age groups of age-related cataract tissues. Truscott found that oxidative stress produced in the early stage of cataract and the level of H_2_O_2_ in the atrial water of cataract patients increased significantly higher than that of normal people [33]. There was research on age-related cataract using H_2_O_2_ conditions in vitro. Yao et al. [9] and Zhou et al. [10] set 50 *μ*M H_2_O_2_ as oxidative stress conditions in treating SRA01/04 cells, finding that apoptosis and autophagy induced by H_2_O_2_ are in a time-dependent manner. In vitro studies have shown that the same concentration of hydrogen peroxide in the lens of cataract patients can lead to lens epithelial cell apoptosis and lens opacification, which is the same as the pathological manifestations of cataract patients [34]. Therefore, we chose SRA01/04 cells cultured under oxidative stress as the age-related cataract model in the present study and found that let-7c-3p was downregulated in SRA01/04 cells and age-related cataract tissues (>65 years old). Meanwhile, let-7c-3p could suppress autophagy and apoptosis under oxidative stress, which might contribute to age-related cataract.

miRNAs are involved in nearly every fields of cellular processes like proliferation, apoptosis, metabolism, and autophagy [22, 23, 25, 26]. So many diseases have been investigated to be associated with miRNA expression [35, 36]. And multiple miRNAs have a terrific relationship with the formation and progression of age-related cataract. For example, let-7 family miRNAs are a group of 21-nucleotide-length miRNAs with 13 members [37] and let-7b has been declared to induce apoptosis of LECs through targeting Lgr4 [26]. Recently, some researchers had proved that let-7b play an important role in the progression of cataract, but it has not explained the retail mechanisms of let-7c in this process, except the let-7c expression levels and either the severity of lens opacity or the patient age. The let-7c has a crucial role in the process of autophagy [13, 31, 38], and a recent study has found that autophagy can act a very important role in keeping the transparency of lenses [13]. Simultaneously, the effect of let-7c-3p during age-related cataract formation is unknown. In our research, we found that let-7c-3p was downregulated in SRA01/04 cells under oxidative stress, suggesting that let-7c-3p might be involved in the formation of human age-related cataract. Apoptosis was found inhibited by let-7c-3p in SRA01/04 cells under oxidative stress. The ratio of LC3B II and LC3B I and the immunofluorescence assay revealed that let-7c-3p attenuated autophagy induced by oxidative stress.

ATG3 is considered as a vital modulator of autophagy in mediating mitochondrial homeostasis [32] and has been reported to be involved in the regulation of autophagy and cell viability [39]. In the present research, we found that oxidative stress induced the increase of ATG3, and ATG3 could facilitate autophagy in SRA01/04 cells under oxidative stress. The bioinformatics database and luciferase reporter assay indicated that let-7c-3p could bind the 3′UTR region of ATG3 mRNA directly to regulate autophagy in SRA01/04 cells. However, the correlation of autophagy and apoptosis in the formation of age-related cataract requires further research. To sum up, we indicate that let-7c-3p regulates apoptosis and ATG3-mediated autophagy in LECs under oxidative stress, which implies that let-7c-3p might be a novel target for age-related cataract therapy.

## 5. Conclusion

This study is aimed at investigating the effects of let-7c-3p in lens epithelial cells (LECs) in vitro and involved the process of autophagy and apoptosis. Let-7c-3p inhibits autophagy by targeting ATG3 in LECs in age-related cataract.

## Data Availability

The data used to support the findings of this study are included within the article.
